# Controversies in Neurology: why monoamine oxidase B inhibitors could be a good choice for the initial treatment of Parkinson's disease

**DOI:** 10.1186/1471-2377-11-112

**Published:** 2011-09-22

**Authors:** Matthias Löhle, Heinz Reichmann

**Affiliations:** 1 Department of Neurology, University Hospital Carl Gustav Carus, Dresden University of Technology, Fetscherstrasse 74, 01307 Dresden, Germany

## Abstract

**Background:**

Early initiation of pharmacotherapy in Parkinson's disease (PD) is nowadays widely advocated by experts since the delay of treatment has shown to be associated with a significant deterioration of health related quality of life in affected patients. Due to marked advances in PD treatment during the last decades, physicians are nowadays fortunately equipped with a variety of substances that can effectively ameliorate emerging motor symptoms of the disease, among them levodopa, dopamine agonists and monoamine oxidase type B (MAO-B) inhibitors. Despite numerous drug intervention trials in early PD, there is however still ongoing controversy among neurologists which substance to use for the initial treatment of the disease.

**Discussion:**

In multiple studies, MAO-B inhibitors, such as selegiline and rasagiline, have shown to provide mild symptomatic effects, delay the need for levodopa, and to reduce the incidence of motor fluctuations. Although their symptomatic efficacy is inferior compared to dopamine agonists and levodopa, MAO-B inhibitors undoubtedly have fewer side effects and are easy to administer. In contrary to their competitors, MAO-B inhibitors may furthermore offer a chance for disease modification, which so far remains a major unmet need in the management of PD and eventually makes them ideal candidates for the early treatment of the disease.

**Summary:**

MAO-B inhibitors may constitute a preferable therapeutic option for early PD, mainly due to their favourable safety profile and their putative neuroprotective capabilities. Since the symptomatic effects of MAO-B inhibitors are comparatively mild, dopamine agonists and levodopa should however be considered for initial treatment in those PD patients, in whom robust and immediate symptomatic relief needs to be prioritized.

## Background

Parkinson's disease (PD) is the second most common neurodegenerative disease after Alzheimer's disease and estimated to affect about 1-2% of the population over 65 years [1]. Early treatment of emerging symptoms is nowadays widely recommended, since PD patients left untreated at diagnosis show a significant worsening in their health related quality of life in comparison to those patients in whom treatment is initiated at or soon after diagnosis [2]. Furthermore, it has been proposed that early treatment of PD may be associated with a correction of basal ganglia functional abnormalities caused by dopaminergic cell loss and dopamine deficiency and could therefore support intrinsic physiological compensatory mechanisms during the course of the disease [3].

Once PD has been diagnosed, physicians eventually find themselves faced by the question, which substance to choose for the initial treatment of the disease. Fortunately, we are nowadays provided with a variety of substances that can effectively counteract the motor symptoms of the disease, among them levodopa, dopamine agonists and monoamine oxidase type B (MAO-B) inhibitors. In the following synopsis, we would like to address why MAO-B inhibitors could be preferred in this situation and may constitute the best option for the initial treatment of PD.

## Discussion

### MAO-B inhibitors provide mild symptomatic effects in early PD

Several large-scale, randomized placebo-controlled clinical trials have demonstrated that treatment with MAO-B inhibitors leads to a symptomatic amelioration of early PD [4-9]. For example, daily treatment with 10 mg selegiline (*N*-Propargylmethamphetamine) in the Deprenyl and Tocopherol Antioxidative Therapy of Parkinsonism (DATATOP) study was associated with an improvement of about 3 points on the total Unified Parkinson's Disease Rating Scale (UPDRS) and 1.7 points on the motor subscale of the UPDRS compared to placebo after 3 months [4]. Symptomatic effects of similar extent have been observed with the second-generation MAO-B inhibitor rasagiline (*N*-Propargyl-1[R]aminoindan), which for example in the TVP-1012 in early monotherapy for PD outpatients (TEMPO) study led to an improvement of about 3 points on the total UPDRS after 3 months at a daily dose of 1 mg per day [5]. Although it can be argued that the symptomatic effects of MAO-B inhibitors may not reach a clinically meaningful magnitude [10] and are clearly inferior in comparison to those seen with modern dopamine agonists [11-14] or even the gold-standard levodopa [15,16], MAO-B inhibitors may still be good candidates for initial PD treatment, since they are able to attenuate the usually mild motor symptoms during the early phase of PD, thereby facilitating the sparing of symptomatically stronger agents for later stages of the disease, when the magnitude of motor impairment ultimately requires more robust symptomatic action. Indeed, multiple studies have successfully demonstrated that early treatment with the MAO-B inhibitors selegiline or lazabemide is capable to delay the need to start with levodopa in PD patients [4,8,9,17-19]. Since long-term levodopa treatment is known to be associated with motor complications, the levodopa-sparing effect of MAO-B inhibitors is likely to be beneficial in the long run, although clinical studies so far failed to prove an antidyskinetic effect of MAO-B inhibition, which may have been due to wide confidence intervals in these studies [20]. However, clinical studies have demonstrated that primary treatment with the MAO-B inhibitor selegiline reduces the incidence of motor fluctuations compared to placebo [21] as well as to levodopa treatment [22]. Similarly, initial monotherapy with the dopamine agonists ropinirole and pramipexole has shown to significantly decrease the incidence of dyskinesias and to a lesser extent also motor fluctuations [23,24]. However, this did not translate into a benefit in terms of overall function or quality of life in comparison to patients that were initially treated with levodopa [23,24], which somewhat argues against a general preference of dopamine agonists over other antiparkinsonian agents in the early stages of PD. In summary, initial treatment with MAO-B inhibitors provides a mild effect on motor symptoms in patients with early PD, delays the need for levodopa, reduces the incidence of motor fluctuations and may furthermore turn out advantageous from a strategic perspective, since it enables physicians to counteract emerging symptoms of early PD without already utilizing the strongest symptomatic options at the very beginning. Due to their superior symptomatic efficacy, dopamine agonists and levodopa should however be considered for initial treatment particularly in those PD patients, who require immediate and robust symptomatic control of their motor symptoms.

### MAO-B inhibitors are well tolerated

When PD patients are subjected to chronic pharmacological treatment for the first time, side effects of the initial therapy will have a major impact on their judgement of antiparkinsonian agents and eventually also be crucial for long-term adherence to PD treatment. Thus, it seems wise to choose an antiparkinsonian agent for initial treatment that provides both symptomatic efficacy and an advantageous side-effect profile. The use of dopamine agonists is often accompanied by adverse events such as nausea, dizziness, somnolence, oedema and hallucinations, which in long-term clinical studies led to substantial drop rates of about 30 to 40% [23,25]. Although treatment with levodopa is considered to be safe and well tolerated, comparative studies between levodopa and dopamine agonists found similar drop rates due to side effects in levodopa treated patients as with dopamine agonists [23,25]. Furthermore, long-term administration of levodopa is known to be associated with motor complications, such as dyskinesia and wearing-off [15,26], which argues against its early use in PD.

On the contrary, clinical studies have demonstrated that MAO-B inhibitors are well tolerated and usually do not cause substantial side effects [20]. Although the use of selegiline has been associated with adverse events like anorexia, nausea, dry mouth, dyskinesia, musculoskeletal injuries, hallucinations, cardiac arrhythmias and orthostatic hypotension, which may be due to an augmentation of dopaminergic activity or amphetamine metabolites of selegiline [27-31], it is important to note that a meta-analysis of placebo-controlled trials found no difference in dropouts due to adverse events between patients treated with selegiline and patients treated with placebo [20]. This observation suggests that side effects of selegiline are not substantial enough to cause patients to end therapy. Especially at higher doses, selegiline treatment may however harbour a potential risk for dietary tyramine-provoked hypertensive crisis [32], since its metabolites metamphetamine and amphetamine are also capable to inhibit peripheral MAO-A [33,34], which is crucial for the metabolism of tyramine [35]. Before initiating selegiline treatment, it is therefore advisable to instruct patients to avoid excessive intake of tyramine-rich foods, such as aged cheese, tap beer, red wine and salami. It should also be noted that in the past there has been some controversy about the safety of selegiline after a study by the Parkinson's Disease Research Group of the United Kingdom had reported excess mortality in patients subjected to a combined treatment with levodopa and selegiline [36], which seemed to affect particularly those individuals with postural hypotension, frequent falls, confusion and dementia [37]. However, later meta-analysis found no significant difference in mortality between patients on MAO-B inhibitors and control patients and instead confirmed their good tolerability [20]. The safety profile of conventional selegiline may moreover be further improved by the use of its orally disintegrating form, which due to improved bioavailability can be administered at lower doses with comparable efficacy and produces lower concentrations of potentially troublesome metamphetamine metabolites by bypassing first-pass metabolism in the liver [38].

In contrast to selegiline, the second-generation MAO-B inhibitor rasagiline is metabolized to aminoindan, which is devoid of an amphetamine-like chemical scaffolding and is thus considered not to have vasoactive properties. Indeed, clinical studies demonstrated good tolerability of rasagiline treatment and found no difference in the frequency of cardiovascular and psychiatric adverse events between rasagiline and placebo treated patients [5,6,39,40]. The excellent side-effect profile of rasagiline has been recently confirmed in the Attenuation of Disease Progression with Azilect Given Once-daily (ADAGIO) trial, which showed no significant differences in adverse events between placebo-treated patients and study participants subjected to daily treatment with 1 mg or 2 mg rasagiline [7]. Moreover, a recently published tyramine challenge study has demonstrated that rasagiline selectively inhibits MAO-B and is not associated with increased tyramine sensitivity at the recommended daily dose of 1 mg [41].

Taken together, data from clinical trials is supportive of good tolerability and safety of MAO-B inhibitors as antiparkinsonian agents, which is a second argument for their use in early PD. Caution should be exercised when administering MAO-B inhibitors together with drugs that increase sympathetic tone, such as ephedrine and phenylephrine, in order to avert the risk of hypertensive crisis. Moreover, concomitant treatment with serotinergic analgesics, such as meperidine, tramadol, methadone and propoxyphene, should be generally avoided in order to prevent a serotonin syndrome [35]. Caution is also advised when administering MAO-B inhibitors together with antidepressants that increase serotonin levels, such as selective serotonin reuptake inhibitors, imipraminics and serotonin-norepinephrine uptake inhibitors, although available studies suggest that the risk to cause serotonin toxicity due to concomitant treatment with antidepressants is rather low [42,43].

### MAO-B inhibitors are easy to use

Whenever long-term administration of pharmacological agents becomes necessary, physicians need to ensure that the dosing regimen is not too complex in order to be followed by patients, since non-compliance may result in early discontinuation, underuse, overuse and irregular ingestion of medication. Previous smaller studies have demonstrated that therapy adherence in PD patients is suboptimal and approximates that reported in other chronic diseases [44,45]. In a larger European multi-center study initiated by Donald Grosset, we have been recently examining drug adherence in 112 PD patients for 4 weeks using electronic monitoring bottles, which recorded the date and time of cap opening [46]. In this study, 12.5% of PD patients took less than 80% of prescribed medication, which was associated with significantly poorer motor function and mobility [46]. Although overall total adherence to PD treatment was high, we observed considerable variation of medication intake between days and often irregular timing of medication intake, which argues against an early use of levodopa in PD, since inaccurate timing of levodopa intake may result in pulsatile dopaminergic stimulation and accelerate the development of motor complications [47]. Indeed, patients with motor fluctuations in our study had significantly lower total and timing adherence than nonfluctuators [46]. On the contrary, once daily drugs had significantly better adherence when compared with drugs prescribed more frequently, which strongly supports the usage of novel extended-release formulations of dopamine agonists or of MAO-B inhibitors, whereas the latter are additionally capable to provide a more sustained and continuous stimulation of the post-synaptic dopaminergic receptors by prolonging the half-life of dopamine in the basal ganglia.

Another possible obstacle to regular medication intake in PD patients may be dysphagia, which although more common in advanced stages can also be observed in early PD [48,49]. In patients with swallowing difficulties, PD treatment may be initiated with the orally disintegrating form of selegiline, which is nowadays available on the market as Otrasel^®^, Xilopar^® ^and Zelapar^®^. In contrast to conventional selegiline and other antiparkinsonian agents, orally disintegrating selegiline is refined by lyophilisation using Zydis technology^® ^[50], which ensures rapid dissolving in the mouth and facilitates pregastral (predominantly buccal) absorption. Patient preference for orally disintegrating selegiline has been evaluated in a single-dose, randomised, two-way crossover study in 148 PD patients, in which about 30% were suffering from swallowing difficulties [51]. In this study, PD patients who had been previously treated with 10 mg of conventional selegiline as adjunctive therapy were transiently switched to a single dose of 5 mg of orally disintegrating selegiline or a placebo fast-dissolving formulation and asked for their preference. Most patients (61%) liked the novel formulation of selegiline, which was a significantly greater proportion than the null hypothesis of 50% (p < 0.002). Moreover, 67% of the patients with swallowing difficulties preferred orally disintegrating selegiline to conventional selegiline tablets [51]. These results indicate that alongside parenteral forms of dopamine agonists, such as transdermal rotigotine and subcutaneous apomorphine, orally disintegrating selegiline might be a worthwhile option for treatment in those PD patients, in whom swallowing difficulties already constitute a problem at the beginning of the disease.

### MAO-B inhibitors potentially have disease-modifying capabilities

Despite marked advances in symptomatic treatment during recent decades, clinical studies in PD patients have so far failed to provide compelling proof for neuroprotective capabilities in most antiparkinsonian agents [52]. In the absence of disease-modifying therapies, symptomatic treatment can only transiently compensate for the progressive neurodegeneration in the nigrostriatal system and in non-mesencephalic brain regions, which will eventually lead to motor fluctuations, dyskinesias and intolerable disability in most PD patients [53]. Consequently, neuroprotective therapy that slows or even stops disease progression is considered a major unmet medical need in the treatment of PD [54].

Data from preclinical studies has redundantly suggested that MAO-B inhibitors may have neuroprotective properties. For example, previous studies demonstrated that selegiline is able to block the development of 1-methyl-4-phenyl-1,2,3,6-tetrahydropyridine(MPTP)-induced neurotoxicity [55,56] and *in vivo *may protect mesencephalic dopaminergic neurons from glutamate receptor-mediated toxicity [57,58] and from toxicity induced by glutathione depletion [59]. Similar results were also obtained with rasagiline, which was found to increase survival in dopaminergic neurons [60,61] and to protect against neurotoxin-induced apoptotic cell death in SH-SY5Y cells [62,63]. Subsequent studies in animal models of PD also demonstrated *in vivo *that pre-treatment with selegiline or rasagiline is capable to markedly attenuate the behavioural deficits arising from neurotoxic MPTP and 6-hydroxydopamine injections [64,65], thereby nourishing further hope that MAO-B inhibitors would also harbour disease-modifying capabilities in the clinical setting.

One of the earliest clinical studies to examine neuroprotective effects in MAO-B inhibitors has been the Deprenyl (= selegiline) and Tocopherol Antioxidative Therapy of Parkinson (DATATOP) trial, in which 800 patients with early PD were randomized to four treatment arms: 1) tocopherol placebo and selegiline placebo, 2) 2000 IU tocopherol per day and selegiline placebo, 3) tocopherol placebo and 10 mg selegiline per day and 4) 2000 IU tocopherol and 10 mg selegiline per day [4,66]. This study was prematurely terminated after a mean of 12 months when an interim analysis revealed that patients treated with selegiline required levodopa nine months later than individuals who were not randomized to selegiline treatment [66]. Although this delay in the onset of disability necessitating levodopa therapy was tempting to the conclusion that selegiline would be neuroprotective and had also been found in similar studies [9,18], final analysis of the DATATOP study demonstrated that the benefit of selegiline in delaying disability was related to a symptomatic relief of PD [4]. Moreover, long-term observation of the study cohort demonstrated that after an average of 8.2 years the mortality rate was unaffected by selegiline treatment, thus further questioning a putative disease-modifying effect of this substance [67].

More promising results have been found with rasagiline, which has been investigated in two double-blind, placebo-controlled clinical trials, which both utilized the delayed-start design in order to separate potential neuroprotective properties of rasagiline from its symptomatic effects [6,7]. In the TVP-1012 in early monotherapy for PD outpatients (TEMPO) study, 404 previously untreated patients with early PD were randomized to one of three treatment groups: 1) 1 mg rasagiline per day for 1 year, 2) 2 mg rasagiline per day for 1 year or 3) placebo for 6 months followed by 2 mg rasagiline per day for 6 months. The difference between early and delayed treatment on the total UPDRS after one year was about 2 points, which cannot be purely attributed to a symptomatic effect of rasagiline [6]. Moreover, results of an open-label extension of the TEMPO study recently demonstrated that early rasagiline treatment in comparison to delayed treatment provided long-term clinical benefit over years even in the face of treatment with other dopaminergic agents, which can only be explained by an enduring neuroprotective effect of rasagiline or an influence on compensatory mechanisms in early PD [68]. Disease-modifying effects of rasagiline have also been examined in the Attenuation of Disease Progression with Azilect Given Once-daily (ADAGIO) study, which was performed in two phases, each lasting 36 weeks [7]. In the first phase, 1176 subjects were randomly assigned to one of four study groups: rasagiline at a dose of either 1 mg or 2 mg per day (the early-start groups) or corresponding placebo. In the second phase, the early-start groups continued to receive their assigned treatment while subjects in the placebo groups were switched to rasagiline at a dose of 1 mg or 2 mg per day (the delayed-start groups). Three end points had to be met in hierarchical fashion in order to declare positive results, which comprised 1) less worsening in the rate of change in the total UPDRS score between weeks 12 and 36 as compared with placebo, 2) less worsening in the total UPDRS score between baseline and week 72 in the early-start group than in the delayed-start group and 3) non-inferiority in the rate of worsening in the total UPDRS score between weeks 48 and 72 in the early-start group in comparison to the delayed-start group [7]. Rasagiline at a dose of 1 mg per day met all three pre-defined end points, which is consistent with a possible disease-modifying effect of this treatment. Surprisingly, results were negative for patients treated with rasagiline at a daily dose of 2 mg, since this dose failed to meet the second endpoint. However, ADAGIO investigators hypothesized that symptomatic effects of rasagiline 2 mg per day may have masked disease-modifying effects in this population of patients with very mild disease, which was supported by a post-hoc subgroup analysis revealing that the second endpoint was indeed met in those subjects, who were in the highest quartile of UPDRS scores at baseline [7]. Despite uncertainties arising from the ADAGIO study, the functional significance of its results and putative general concerns with the delayed-start design [69], it is important to note that the clinical data on neuroprotective effects of rasagiline are so far unrivalled in comparison to trials with other antiparkinsonian agents. Although previous studies have also suggested neuroprotective properties for levodopa [15] and for dopamine agonists [70,71] by using neuroimaging biomarkers as surrogate endpoint for disease progression, the significance of these findings remains rather questionable due to methodological difficulties and poor correlation between radiotracer imaging and the eventual clinical outcome [52,72]. On the contrary, the long-term clinical benefit that has been observed in the open-label extension of TEMPO eventually provides at least some scientific rationale for assuming disease-modifying effects of rasagiline in early PD, which may provide another good reason to favour MAO-B inhibitors for the initial treatment of PD.

## Summary

Despite ongoing controversies among neurologists on how to initiate treatment in early PD there are compelling arguments for MAO-B inhibitors to be preferred in this situation. In clinical studies, they have shown to ameliorate symptoms of the disease, to delay the need for levodopa and to reduce the incidence of motor fluctuations. Furthermore, MAO-B inhibitors come with no substantial side effects and are easy to administer, which facilitates long-term adherence of patients to the therapy. In contrary to other antiparkinsonian agents such as levodopa and dopamine agonists, there is moreover growing and so far unrivalled clinical evidence for potential disease-modifying effects of MAO-B inhibitors in PD, which may eventually only be fully exploited by an early application from the very beginning of the disease. On the contrary, it needs to be acknowledged that the symptomatic efficacy of MAO-B inhibitors is clearly inferior to those of dopamine agonists and levodopa, which should be particularly considered if immediate and robust control of motor symptoms is warranted. In the future, head-to-head comparison of antiparkinsonian agents (such as in the ongoing PD MED trial) may allow an unbiased and patient-orientated assessment of the risk-benefit relationship in individual PD medications and eventually facilitate the decision on how to initiate treatment in early PD.

## Competing interests

ML has been funded by a research grant of the Center for Regenerative Therapies Dresden and received honoraria for presentations from Boehringer Ingelheim and GlaxoSmithKline. HR has was acting on Advisory Boards and received honoraria and research funds from Abbott, Bayer HealthCare, Boehringer Ingelheim, Cephalon, Desitin, GlaxoSmithKline, Merck Serono, Novartis, Orion, Pfizer, Teva/Lundbeck, UCB Pharma and Valeant.

## Authors' contributions

ML drafted the manuscript. HR critically revised the manuscript. Both authors read and approved the final manuscript.

## Pre-publication history

The pre-publication history for this paper can be accessed here:

http://www.biomedcentral.com/1471-2377/11/112/prepub

## References

[B1] Alves G Forsaa EB Pedersen KF Dreetz Gjerstad M Larsen JP Epidemiology of Parkinson's disease J Neurol 2008 255 Suppl 5 18 32 18787879 10.1007/s00415-008-5004-3

[B2] Grosset D Taurah L Burn DJ MacMahon D Forbes A Turner K Bowron A Walker R Findley L Foster O A multicentre longitudinal observational study of changes in self reported health status in people with Parkinson's disease left untreated at diagnosis J Neurol Neurosurg Psychiatry 2007 78 5 465 469 17098846 10.1136/jnnp.2006.098327 PMC2117846

[B3] Schapira AH Obeso J Timing of treatment initiation in Parkinson's disease: a need for reappraisal? Ann Neurol 2006 59 3 559 562 10.1002/ana.20789 16489611

[B4] Parkinson Study Group Effects of tocopherol and deprenyl on the progression of disability in early Parkinson's disease N Engl J Med 1993 328 3 176 183 8417384 10.1056/NEJM199301213280305

[B5] Parkinson Study Group A controlled trial of rasagiline in early Parkinson disease: the TEMPO Study Arch Neurol 2002 59 12 1937 1943 10.1001/archneur.59.12.1937 12470183

[B6] Parkinson Study Group A controlled, randomized, delayed-start study of rasagiline in early Parkinson disease Arch Neurol 2004 61 4 561 566 10.1001/archneur.61.4.561 15096406

[B7] Olanow CW Rascol O Hauser R Feigin PD Jankovic J Lang A Langston W Melamed E Poewe W Stocchi F A double-blind, delayed-start trial of rasagiline in Parkinson's disease N Engl J Med 2009 361 13 1268 1278 10.1056/NEJMoa0809335 19776408

[B8] Allain H Pollak P Neukirch HC Symptomatic effect of selegiline in de novo Parkinsonian patients. The French Selegiline Multicenter Trial Mov Disord 1993 8 Suppl 1 S36 40 8302306 10.1002/mds.870080508

[B9] Palhagen S Heinonen EH Hagglund J Kaugesaar T Kontants H Maki-Ikola O Palm R Turunen J Selegiline delays the onset of disability in de novo parkinsonian patients. Swedish Parkinson Study Group Neurology 1998 51 2 520 525 9710028 10.1212/wnl.51.2.520

[B10] Schrag A Sampaio C Counsell N Poewe W Minimal clinically important change on the unified Parkinson's disease rating scale Mov Disord 2006 21 8 1200 1207 10.1002/mds.20914 16673410

[B11] Parkinson Study Group Safety and efficacy of pramipexole in early Parkinson disease. A randomized dose-ranging study Jama 1997 278 2 125 130 9214527 10.1001/jama.1997.03550020057038

[B12] Adler CH Sethi KD Hauser RA Davis TL Hammerstad JP Bertoni J Taylor RL Sanchez-Ramos J O'Brien CF Ropinirole for the treatment of early Parkinson's disease. The Ropinirole Study Group Neurology 1997 49 2 393 399 9270567 10.1212/wnl.49.2.393

[B13] Shannon KM Bennett JP Jr Friedman JH Efficacy of pramipexole, a novel dopamine agonist, as monotherapy in mild to moderate Parkinson's disease. The Pramipexole Study Group Neurology 1997 49 3 724 728 9305331 10.1212/wnl.49.3.724

[B14] Parkinson Study Group A controlled trial of rotigotine monotherapy in early Parkinson's disease Arch Neurol 2003 60 12 1721 1728 10.1001/archneur.60.12.1721 14676046

[B15] Fahn S Oakes D Shoulson I Kieburtz K Rudolph A Lang A Olanow CW Tanner C Marek K Levodopa and the progression of Parkinson's disease N Engl J Med 2004 351 24 2498 2508 15590952 10.1056/NEJMoa033447

[B16] Hauser RA Auinger P Oakes D Levodopa response in early Parkinson's disease Mov Disord 2009 24 16 2328 2336 19908302 10.1002/mds.22759

[B17] Parkinson Study Group Effect of lazabemide on the progression of disability in early Parkinson's disease Ann Neurol 1996 40 1 99 107 10.1002/ana.410400116 8687199

[B18] Tetrud JW Langston JW The effect of deprenyl (selegiline) on the natural history of Parkinson's disease Science 1989 245 4917 519 522 10.1126/science.2502843 2502843

[B19] Myllyla VV Sotaniemi KA Vuorinen JA Heinonen EH Selegiline as initial treatment in de novo parkinsonian patients Neurology 1992 42 2 339 343 1736162 10.1212/wnl.42.2.339

[B20] Ives NJ Stowe RL Marro J Counsell C Macleod A Clarke CE Gray R Wheatley K Monoamine oxidase type B inhibitors in early Parkinson's disease: meta-analysis of 17 randomised trials involving 3525 patients BMJ 2004 329 7466 593 10.1136/bmj.38184.606169.AE 15310558 PMC516655

[B21] Myllyla VV Sotaniemi KA Hakulinen P Maki-Ikola O Heinonen EH Selegiline as the primary treatment of Parkinson's disease--a long-term double-blind study Acta Neurol Scand 1997 95 4 211 218 10.1111/j.1600-0404.1997.tb00101.x 9150811

[B22] Caraceni T Musicco M Levodopa or dopamine agonists, or deprenyl as initial treatment for Parkinson's disease. A randomized multicenter study Parkinsonism Relat Disord 2001 7 2 107 114 10.1016/S1353-8020(00)00023-7 11248591

[B23] Rascol O Brooks DJ Korczyn AD De DPP Clarke CE Lang AE A five-year study of the incidence of dyskinesia in patients with early Parkinson's disease who were treated with ropinirole or levodopa N Engl J Med 2000 342 1484 1491 10.1056/NEJM200005183422004 10816186

[B24] Parkinson Study Group CALM Cohort Investigators Long-term effect of initiating pramipexole vs levodopa in early Parkinson disease Arch Neurol 2009 66 5 563 570 19433655 10.1001/archneur.66.1.nct90001

[B25] Holloway RG Shoulson I Fahn S Kieburtz K Lang A Marek K McDermott M Seibyl J Weiner W Musch B Pramipexole vs levodopa as initial treatment for Parkinson disease: a 4-year randomized controlled trial Arch Neurol 2004 61 7 1044 1053 15262734 10.1001/archneur.61.7.1044

[B26] Poewe WH Lees AJ Stern GM Low-dose L-dopa therapy in Parkinson's disease: a 6-year follow-up study Neurology 1986 36 11 1528 1530 3762973 10.1212/wnl.36.11.1528

[B27] Martin WR Sloan JW Sapira JD Jasinski DR Physiologic, subjective, and behavioral effects of amphetamine, methamphetamine, ephedrine, phenmetrazine, and methylphenidate in man Clin Pharmacol Ther 1971 12 2 245 258 5554941 10.1002/cpt1971122part1245

[B28] Churchyard A Mathias CJ Boonkongchuen P Lees AJ Autonomic effects of selegiline: possible cardiovascular toxicity in Parkinson's disease J Neurol Neurosurg Psychiatry 1997 63 2 228 234 10.1136/jnnp.63.2.228 9285463 PMC2169684

[B29] Yasar S Goldberg JP Goldberg SR Are metabolites of l-deprenyl (selegiline) useful or harmful? Indications from preclinical research J Neural Transm Suppl 1996 48 61 73 8988462 10.1007/978-3-7091-7494-4_6

[B30] Yasar S Justinova Z Lee SH Stefanski R Goldberg SR Tanda G Metabolic transformation plays a primary role in the psychostimulant-like discriminative-stimulus effects of selegiline [(R)-(-)-deprenyl] J Pharmacol Exp Ther 2006 317 1 387 394 16352699 10.1124/jpet.105.096263

[B31] Gill JR Jr Masson DT Bartter FC Effects of hydroxyamphetamine (paredrine) on the function of the sympathetic nervous system in normotensive subjects J Pharmacol Exp Ther 1967 155 2 288 295 6025520

[B32] Schulz R Antonin KH Hoffmann E Jedrychowski M Nilsson E Schick C Bieck PR Tyramine kinetics and pressor sensitivity during monoamine oxidase inhibition by selegiline Clin Pharmacol Ther 1989 46 5 528 536 10.1038/clpt.1989.181 2510962

[B33] Mantle TJ Tipton KF Garrett NJ Inhibition of monoamine oxidase by amphetamine and related compounds Biochem Pharmacol 1976 25 18 2073 2077 10.1016/0006-2952(76)90432-9 985546

[B34] Suzuki O Hattori H Asano M Oya M Katsumata Y Inhibition of monoamine oxidase by d-methamphetamine Biochem Pharmacol 1980 29 14 2071 2073 10.1016/0006-2952(80)90493-1 6773528

[B35] Chen JJ Swope DM Dashtipour K Comprehensive review of rasagiline, a second-generation monoamine oxidase inhibitor, for the treatment of Parkinson's disease Clin Ther 2007 29 9 1825 1849 10.1016/j.clinthera.2007.09.021 18035186

[B36] Lees AJ Comparison of therapeutic effects and mortality data of levodopa and levodopa combined with selegiline in patients with early, mild Parkinson's disease. Parkinson's Disease Research Group of the United Kingdom BMJ 1995 311 7020 1602 1607 8555803 10.1136/bmj.311.7020.1602 PMC2551499

[B37] Ben-Shlomo Y Churchyard A Head J Hurwitz B Overstall P Ockelford J Lees AJ Investigation by Parkinson's Disease Research Group of United Kingdom into excess mortality seen with combined levodopa and selegiline treatment in patients with early, mild Parkinson's disease: further results of randomised trial and confidential inquiry BMJ 1998 316 7139 1191 1196 9583926 10.1136/bmj.316.7139.1191 PMC28519

[B38] Löhle M Storch A Orally disintegrating selegiline for the treatment of Parkinson's disease Expert Opin Pharmacother 2008 9 16 2881 2891 10.1517/14656566.9.16.2881 18937619

[B39] Rascol O Brooks DJ Melamed E Oertel W Poewe W Stocchi F Tolosa E Rasagiline as an adjunct to levodopa in patients with Parkinson's disease and motor fluctuations (LARGO, Lasting effect in Adjunct therapy with Rasagiline Given Once daily, study): a randomised, double-blind, parallel-group trial Lancet 2005 365 9463 947 954 10.1016/S0140-6736(05)71083-7 15766996

[B40] Parkinson Study Group A randomized placebo-controlled trial of rasagiline in levodopa-treated patients with Parkinson disease and motor fluctuations: the PRESTO study Arch Neurol 2005 62 2 241 248 15710852 10.1001/archneur.62.2.241

[B41] Goren T Adar L Sasson N Weiss YM Clinical pharmacology tyramine challenge study to determine the selectivity of the monoamine oxidase type B (MAO-B) inhibitor rasagiline J Clin Pharmacol 2010 50 12 1420 1428 10.1177/0091270010369674 20445015

[B42] Perez-Lloret S Rascol O Safety of rasagiline for the treatment of Parkinson's disease Expert Opin Drug Saf 2011 10 4 633 643 10.1517/14740338.2011.573784 21453201

[B43] Richard IH Kurlan R Tanner C Factor S Hubble J Suchowersky O Waters C Serotonin syndrome and the combined use of deprenyl and an antidepressant in Parkinson's disease. Parkinson Study Group Neurology 1997 48 4 1070 1077 9109902 10.1212/wnl.48.4.1070

[B44] Leopold NA Polansky M Hurka MR Drug adherence in Parkinson's disease Mov Disord 2004 19 5 513 517 10.1002/mds.20041 15133814

[B45] Grosset KA Bone I Grosset DG Suboptimal medication adherence in Parkinson's disease Mov Disord 2005 20 11 1502 1507 10.1002/mds.20602 16037924

[B46] Grosset D Antonini A Canesi M Pezzoli G Lees A Shaw K Cubo E Martinez-Martin P Rascol O Negre-Pages L Adherence to antiparkinson medication in a multicenter European study Mov Disord 2009 24 6 826 832 10.1002/mds.22112 19191340

[B47] Stocchi F The hypothesis of the genesis of motor complications and continuous dopaminergic stimulation in the treatment of Parkinson's disease Parkinsonism Relat Disord 2009 15 Suppl 1 S9 S15 19131046 10.1016/S1353-8020(09)70005-7

[B48] Nilsson H Ekberg O Olsson R Hindfelt B Quantitative assessment of oral and pharyngeal function in Parkinson's disease Dysphagia 1996 11 2 144 150 10.1007/BF00417905 8721074

[B49] Potulska A Friedman A Krolicki L Spychala A Swallowing disorders in Parkinson's disease Parkinsonism Relat Disord 2003 9 6 349 353 10.1016/S1353-8020(03)00045-2 12853234

[B50] Virely P Yarwood R Zydis: a novel, fast dissolving dosage form Manuf Chem 1990 61 36 37

[B51] Clarke A Johnson ES Mallard N Corn TH Johnston A Boyce M Warrington S MacMahon DG A new low-dose formulation of selegiline: clinical efficacy, patient preference and selectivity for MAO-B inhibition J Neural Transm 2003 110 11 1257 1271 10.1007/s00702-003-0042-6 14628190

[B52] Löhle M Reichmann H Clinical neuroprotection in Parkinson's disease - still waiting for the breakthrough J Neurol Sci 2010 289 1-2 104 114 10.1016/j.jns.2009.08.025 19772974

[B53] Hely MA Morris JG Reid WG Trafficante R Sydney Multicenter Study of Parkinson's disease: non-L-dopa-responsive problems dominate at 15 years Mov Disord 2005 20 2 190 199 10.1002/mds.20324 15551331

[B54] Olanow CW The scientific basis for the current treatment of Parkinson's disease Annu Rev Med 2004 55 41 60 10.1146/annurev.med.55.091902.104422 14746509

[B55] Cohen G Pasik P Cohen B Leist A Mytilineou C Yahr MD Pargyline and deprenyl prevent the neurotoxicity of 1-methyl-4-phenyl-1,2,3,6-tetrahydropyridine (MPTP) in monkeys Eur J Pharmacol 1984 106 1 209 210 10.1016/0014-2999(84)90700-3 6442232

[B56] Heikkila RE Manzino L Cabbat FS Duvoisin RC Protection against the dopaminergic neurotoxicity of 1-methyl-4-phenyl-1,2,5,6-tetrahydropyridine by monoamine oxidase inhibitors Nature 1984 311 5985 467 469 10.1038/311467a0 6332989

[B57] Mytilineou C Radcliffe P Leonardi EK Werner P Olanow CW L-deprenyl protects mesencephalic dopamine neurons from glutamate receptor-mediated toxicity in vitro J Neurochem 1997 68 1 33 39 8978707 10.1046/j.1471-4159.1997.68010033.x

[B58] Mytilineou C Radcliffe PM Olanow CW L-(-)-desmethylselegiline, a metabolite of selegiline [L-(-)-deprenyl], protects mesencephalic dopamine neurons from excitotoxicity in vitro J Neurochem 1997 68 1 434 436 8978757 10.1046/j.1471-4159.1997.68010434.x

[B59] Mytilineou C Leonardi EK Radcliffe P Heinonen EH Han SK Werner P Cohen G Olanow CW Deprenyl and desmethylselegiline protect mesencephalic neurons from toxicity induced by glutathione depletion J Pharmacol Exp Ther 1998 284 2 700 706 9454817

[B60] Finberg JP Takeshima T Johnston JM Commissiong JW Increased survival of dopaminergic neurons by rasagiline, a monoamine oxidase B inhibitor Neuroreport 1998 9 4 703 707 10.1097/00001756-199803090-00026 9559942

[B61] Goggi J Theofilopoulos S Riaz SS Jauniaux E Stern GM Bradford HF The neuronal survival effects of rasagiline and deprenyl on fetal human and rat ventral mesencephalic neurones in culture Neuroreport 2000 11 18 3937 3941 10.1097/00001756-200012180-00007 11192605

[B62] Maruyama W Akao Y Youdim MB Naoi M Neurotoxins induce apoptosis in dopamine neurons: protection by N-propargylamine-1(R)- and (S)-aminoindan, rasagiline and TV1022 J Neural Transm Suppl 2000 60 171 186 10.1007/978-3-7091-6301-6_11 11205138

[B63] Naoi M Maruyama W Youdim MB Yu P Boulton AA Anti-apoptotic function of propargylamine inhibitors of type-B monoamine oxidase Inflammopharmacology 2003 11 2 175 181 10.1163/156856003765764344 15035819

[B64] Blandini F Armentero MT Fancellu R Blaugrund E Nappi G Neuroprotective effect of rasagiline in a rodent model of Parkinson's disease Exp Neurol 2004 187 2 455 459 10.1016/j.expneurol.2004.03.005 15144871

[B65] Kupsch A Sautter J Gotz ME Breithaupt W Schwarz J Youdim MB Riederer P Gerlach M Oertel WH Monoamine oxidase-inhibition and MPTP-induced neurotoxicity in the non-human primate: comparison of rasagiline (TVP 1012) with selegiline J Neural Transm 2001 108 8-9 985 1009 11716151 10.1007/s007020170018

[B66] Parkinson Study Group Effect of deprenyl on the progression of disability in early Parkinson's disease N Engl J Med 1989 321 20 1364 1371 2509910 10.1056/NEJM198911163212004

[B67] Parkinson Study Group Mortality in DATATOP: a multicenter trial in early Parkinson's disease Ann Neurol 1998 43 3 318 325 10.1002/ana.410430309 9506548

[B68] Hauser RA Lew MF Hurtig HI Ondo WG Wojcieszek J Fitzer-Attas CJ Long-term outcome of early versus delayed rasagiline treatment in early Parkinson's disease Mov Disord 2009 24 4 564 573 10.1002/mds.22402 19086083

[B69] Clarke CE Are delayed-start design trials to show neuroprotection in Parkinson's disease fundamentally flawed? Mov Disord 2008 23 6 784 789 10.1002/mds.21918 18175348

[B70] Parkinson SG Dopamine transporter brain imaging to assess the effects of pramipexole vs levodopa on Parkinson disease progression Jama 2002 287 13 1653 1661 10.1001/jama.287.13.1653 11926889

[B71] Whone AL Watts RL Stoessl AJ Davis M Reske S Nahmias C Lang AE Rascol O Ribeiro MJ Remy P Slower progression of Parkinson's disease with ropinirole versus levodopa: The REAL-PET study Ann Neurol 2003 54 1 93 101 10.1002/ana.10609 12838524

[B72] Ravina B Eidelberg D Ahlskog JE Albin RL Brooks DJ Carbon M Dhawan V Feigin A Fahn S Guttman M The role of radiotracer imaging in Parkinson disease Neurology 2005 64 208 215 10.1212/01.WNL.0000149403.14458.7F 15668415

